# Effects of Blood Flow Restriction Combined with Low-Intensity Resistance Training on Lower-Limb Muscle Strength and Mass in Post-Middle-Aged Adults: A Systematic Review and Meta-Analysis

**DOI:** 10.3390/ijerph192315691

**Published:** 2022-11-25

**Authors:** Hualong Chang, Mengxing Yao, Biao Chen, Yongle Qi, Jianli Zhang

**Affiliations:** 1 College of Physical Education and Health Sciences, Zhejiang Normal University, Jinhua 321004, China; 2 Institute of Human Movement and Sports Engineering, Zhejiang Normal University, Jinhua 321004, China

**Keywords:** blood flow restriction, adults, randomized controlled trial

## Abstract

We studied the effect of blood flow restriction (BFR) combined with low-intensity resistance training (LIRT) on lower-limb muscle strength and mass in post-middle-aged adults. The PubMed, OVID, ProQuest, Cochrane Library, EMBASE, Web of Science, and Scopus databases were used to obtain randomized controlled trials, and the effects of BFR and LIRT (BFRt) on muscle strength and mass in adults were examined. The Cochrane risk of bias tool assessed bias in the included trials. The combined effects of BFR and LIRT (BFRt) were calculated by meta-analysis, the association between muscle strength/mass and interventions was determined by meta-regression, and beneficial variables of intervention were explored by subgroup analysis. A total of 11 articles were included in the meta-analysis. The combined effects showed that BFRt significantly improved lower extremity muscle strength but not muscle mass gain. Meta-regression analysis indicated that the effect of BFRt on changes in muscle strength was correlated with frequency of the intervention. Subgroup analysis revealed that BFRt achieved greater muscle strength gains than normal activity, LIRT, and similar muscle strength gains compared to high-intensity resistance training. The increased muscle strength after BFRt was noticed with a frequency of three times a week, but not with a frequency of two times a week, and the difference between these subgroups was statistically significant. Our findings indicate that BFRt can increase lower-limb muscle strength in post-middle-aged adults. Frequency of intervention is a key variable; particularly, a schedule of three times a week is effective in improving muscle strength.

## 1. Introduction

The world’s elderly population is expected to exceed 1.5 billion by 2050, with one senior citizen for every six people. According to the World Population Prospects released by the United Nations, the global aging trend will intensify further [1]. Older adults may experience some difficulties in their physical functions, balance, and daily activities [2,3,4]. In addition, the increasing aging population correspondingly increases the financial expenditure of a nation, which can cause financial burden [5]. Therefore, maintaining a good of quality of life, physical function, and mental health among older adults is crucial. As such, these issues are gaining an increased attention from scientists and societies.

A series of continuous movements from sitting to standing to walking is essential physical activity for older adults. The degradation or loss of this ability leads to the elderly’s inability to live a normal life or can even result in death [6]. Behaviors such as sitting and standing, climbing stairs, and walking are typically affected by the lower-limb muscle strength. Reduction in lower-limb muscle strength and muscle mass in the elderly increases the probability of falling, death, and other risks [3,4]. In addition, the decline in muscle strength is also a direct cause of the decline in quality of life and bodily function [7,8]. Therefore, improving lower-limb muscle strength in the elderly or post-middle-aged adults is an urgent problem that must be explored.

Exercise can increase the bone mass and improve the muscle function of the elderly while delaying muscle atrophy, osteoporosis, and other problems [9,10]. The American College of Sports Medicine (ACSM) has advised that substantial muscle growth occurs when participants exercise at a moderate or high intensity [11]. However, moderate-to-high-intensity physical exercise can also cause muscle damage and subjective discomfort in adults [12,13], whereas low-intensity resistance training (LIRT) is reported to be safe for older adults [14]. Furthermore, high-intensity resistance training (HIRT) is often not feasible for elderly people suffering from skeletal muscle injuries [15,16]. Blood flow restriction (BFR), which was discovered by Japanese scholars and is also known as KAATSU training, is a training method that restricts blood flow of a certain limb by placing a force band, cuff, etc. on the proximal end of the limb [17]. Studies have shown that the BFR method combined with LIRT (BFRt) does not cause any adverse effects [18] and can produce similar muscle strength gains as HIRT [19,20]. In addition, it does not negatively affect arterial stiffness or humeral coagulation factors in older adults [21]. However, other studies have shown that BFRt tends to result in less muscle strength gain than HIRT that has been conducted for 12 weeks of resistance training, twice a week [22].

Furthermore, certain recent meta-analyses have also concluded that BFRt tends to result in less muscle strength gain than HIRT in older adults [23,24]. In contrast, the results of other meta-analyses showed that BFRt produced the same muscle strength gain as HIRT in older adults. [25,26]. These seemingly contradictory results in research studies and meta-analyses may be due to differences in the intervention protocol that was applied. From the perspective of intervention protocols, only one meta-analysis has yet analyzed the influence of exercise variables (exercise mode, intensity, frequency, and duration) on lower-limb muscle strength and muscle function in older adults. This meta-analysis indicated that training duration may be the key variable that positively correlates with muscle strength gain in older adults. However, the included trials in this meta-analysis comprised multiple forms of exercise combined with BFR, and findings that emphasize the benefits of BFR specifically combined with LIRT are limited [23]. In addition, the influence of training frequency combined with BFR on muscle strength/mass has not been addressed in middle-aged adults. Therefore, our objective was to compare the effects of BFRt on muscle strength and mass. We then further performed regression analyses on different training variables, followed by subgroup analyses to understand how these variables potentially influence the beneficial effects of resistance training on gaining muscle strength and mass in post-middle-aged adults.

## 2. Materials and Methods

The systematic review was performed in accordance with the latest guidelines of preferred reporting items for systematic reviews and meta-analyses (PRISMA) [27]. This study was also registered with PROSPERO (CRD42022364670).

### 2.1. Search Strategy

The whole literature retrieval process was conducted by two independent searchers. The search was conducted through the PubMed, OVID, ProQuest, Cochrane Library, EMBASE, Web of Science, and Scopus databases. There was no time limit for the article, and this search was conducted until 19 January 2022. We independently searched each database using the following keyword searches: “Blood Flow Restriction” or “other deformations” AND “Aged” or “other deformations” AND “Randomized Controlled Trial”, or “other deformations.” Consider PubMed, for example, for which the search terms used were: (“Blood Flow Restriction Therapy”[Mesh] OR BFR Therapy[Title/Abstract] OR BFR Therapies[Title/Abstract] OR Therapy, BFR[Title/Abstract] OR Blood Flow Restriction[Title/Abstract] OR KAATSU[Title/Abstract] OR BFRt[Title/Abstract] OR restricted leg blood flow[Title/Abstract] OR restricted leg muscle blood flow[Title/Abstract] OR blood flow occlusion[Title/Abstract] OR blood flow restricted clastic band training[Title/Abstract] OR Occlusion training[Title/Abstract] OR occluded blood flow[Title/Abstract] OR restricted blood flow[Title/Abstract] OR vascular re-striction[Title/Abstract] OR vascular occlusion[Title/Abstract]) AND (“Aged”[Mesh] OR Elderly[Title/Abstract] OR older people[Title/Abstract] OR older adults[Title/Abstract] OR aging[Title/Abstract] OR senior[Title/Abstract] OR old subjects[Title/Abstract] OR elder[Title/Abstract]) AND (randomized controlled trial[Publication Type] OR randomized[Title/Abstract] OR placebo[Title/Abstract] OR RCT[Title/Abstract] OR random* [Title/Abstract] OR triple blind*[Title/Abstract] OR clinical trial[Title/Abstract] OR allocation[Title/Abstract] OR single blind[Title/Abstract] OR double blind[Title/Abstract] OR Randomized Controlled Trials as Topic[Title/Abstract]). In addition, we manually searched for the relevant articles from the reference list of previously published articles.

### 2.2. Inclusion and Exclusion Criteria

Selection criteria were established according to the participant, intervention, comparison group, outcome, and study type (PICOS).

Prior to inclusion, the titles and abstracts of the searched articles were screened for relevance. Then, full texts of the articles were obtained and reviewed for the inclusion criteria. In order to determine which articles should be included in this study, we adhered the following inclusion criteria: (1) participants: elderly or post-middle-aged adults (age > 50 years); (2) intervention group: blood flow restriction combined with low-intensity resistance training and compared with control group (without blood flow restriction); (3) outcome: muscle strength or muscle mass; (4) study type: randomized controlled trials (RCTs); and (5) blood flow restriction and muscle strength assessment for the lower body extremities. We excluded studies according to these criteria: (1) people with cancer, diabetes, cardiovascular disease, or incapacitation; (2) the outcome index of the article is not the final value and cannot be converted or calculated; (3) the training contents of the experimental group and the control group were different; and (4) trials of a drug supplementation that affects muscle size and strength.

### 2.3. Data Extraction

Data extracted from each included study were as follows: (1) first author name, publishing year, country; (2) characteristics of participants (number, age, gender); (3) characteristics of exercise intervention (intensity, frequency, duration, mode); (4) BFR details (cuff pressure, cuff width, etc.); and (5) methods used to determine the muscle strength and/or muscle mass.

### 2.4. Quality Assessment

Two independent researchers used the Cochrane risk of bias assessment tool to evaluate the selection bias, performance bias, detection bias, attrition bias, reporting bias, and other biases [28]. Any disputes between the parties were negotiated or referred to a corresponding author.

### 2.5. Statistical Analysis

We used meta-analysis software Review Manager 5.4 and Stata 12.0. Due to significant differences between the measurement tools and units of each outcome index, the standardized mean difference (SMD) was used in order to calculate the effect size. Additionally, the mean and standard deviation (SD) of each group after training were used to calculate the study data. If the data provided in the literature were not in the form of mean and SD, the standard errors (SE) and confidence intervals (CI) were calculated into mean and SD by the standard formula. If the data provided in the paper were presented as graphs, we first determined whether the data in the graphs were reported as mean and SD; if not, we extracted data via the Web Plot Digitizer and then converted them into the required format using formulas. After data extraction, the data were imported into Review Manager 5.4 software and processed in continuous variable mode. Meta-regression was performed with Stata 12.0 on training frequency, training duration, cuff pressure, exercise mode, and comparator intervention group in order to explore the source of heterogeneity. Subgroups were used for the purposes of further analysis of heterogeneous factors. Comparisons were performed between experimental (BFRt) trial and control trial (HIRT, LIRT, or normal activity without BFR).

## 3. Results

### 3.1. Search Results

**Figure 1:**
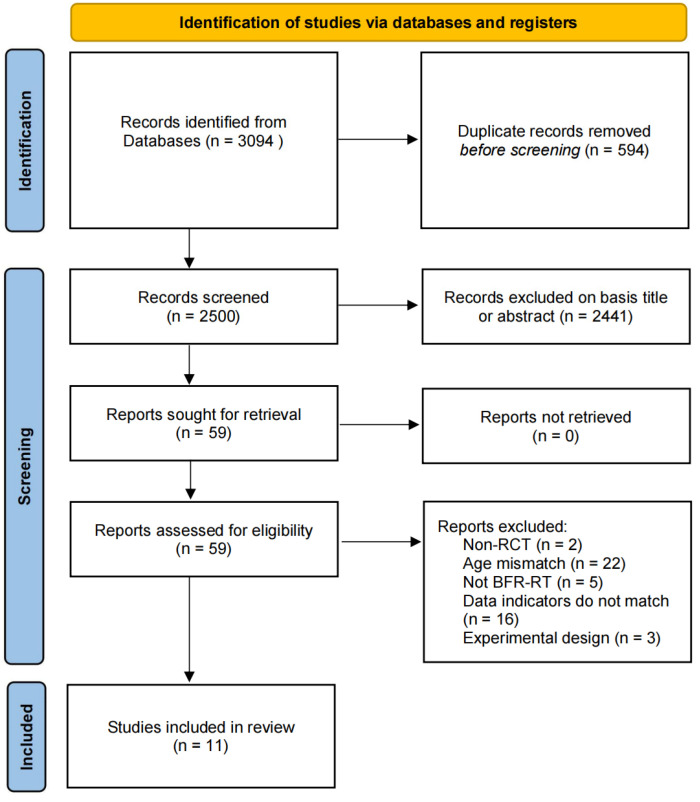
Flowchart of studies selection process.

After screening for titles and abstracts, 3035 articles were excluded from the initial 3094 studies. After evaluation of the full text, a further 48 articles were removed. Finally, after reading the full text, a total of 11 articles met the inclusion criteria of qualitative analysis. The flow chart, which demonstrates this process, is shown in Figure 1.

### 3.2. Characteristics of the Studies

A total of 11 studies (325 participants) were included in this systematic review. Among them, one study recruited only male participants, four studies recruited only female participants, four studies recruited both males and females, and two studies did not report the gender. The sample size of the included studies ranged from 15 to 61 participants in different countries, including five studies conducted in Brazil [19,22,29,30,31], three studies from the United States [32,33,34], and three studies from Japan [35,36,37]. The training frequency for six studies was two days per week, and the training frequency for five studies was three days per week. Cuff pressure was not reported in three of the eleven included studies. Based on exercise intensity, included trials were classified into LIRT (0–49% 1RM) and HIRT (>60% 1RM) with or without BFR, respectively. The training intensity of the BFRt group ranged from 20 to 45% 1RM in all trials. Nine studies were low intensity vs. high intensity [19,22,29,30,31,32,33,34,35], which compared the BFRt group with the non-BFRt high intensity training group of 60–90% 1RM. Two studies were low intensity vs. low intensity [29,37], which compared the BFRt group with the non-BFRt resistance training group of 20–30% 1RM. Six studies compared the BFRt group to a normal activity group [22,29,31,34,35,36]. Furthermore, eleven studies analyzed results related to muscle strength, and six studies analyzed results related to muscle mass. Table 1 summarizes the characteristics of the included articles.

### 3.3. Methodological Quality Assessment of the Included Studies

**Figure 2:**
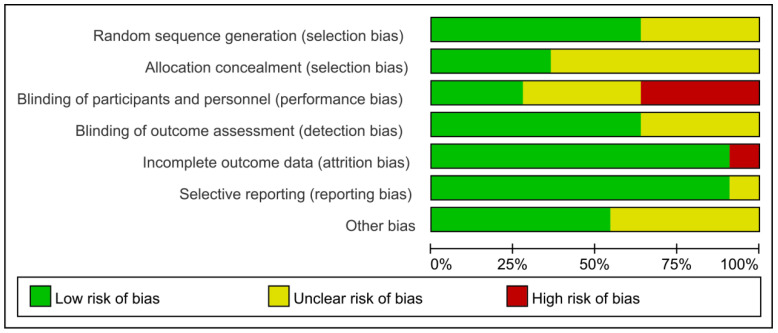
Risk of bias plot.

**Figure 3:**
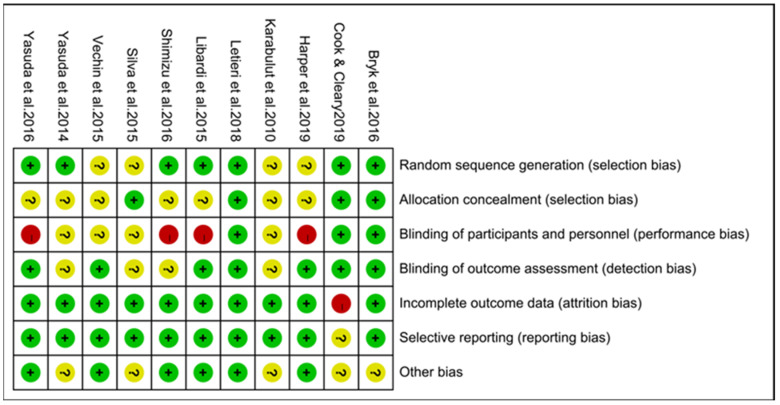
Risk of bias summary plot of included studies [19,22,29,30,31,32,33,34,35,36,37]. Green represents (+) low risk, yellow represents (?) unclear, and red (−) indicates high risk of bias.

The eleven RCTs were included in this study, and bias was determined via the application of Cochrane’s risk of bias assessment tool. As shown in Figure 2 and Figure 3, green represents a low risk, yellow represents unclear, and red represents a high risk of bias. Among the studies, four were listed as unknown risks without a clear explanation of random allocation. Seven studies did not explicitly account for the allocation or concealment and were listed as unknown risks. Three studies blinded participants and researchers. Four studies had implementation bias, with researchers and subjects able to break the blinds. The blinding degree of the remaining studies is unclear. Seven studies described the blindness of the outcome evaluators; further, the blindness of the remaining studies is unclear. Ten studies had complete data reports. One study chose not to report because the data were not commonly used, which was rated a high risk.

### 3.4. Effects of BFRt on Lower-Limb Muscle Strength in Post-Middle-Aged Adults

**Figure 4:**
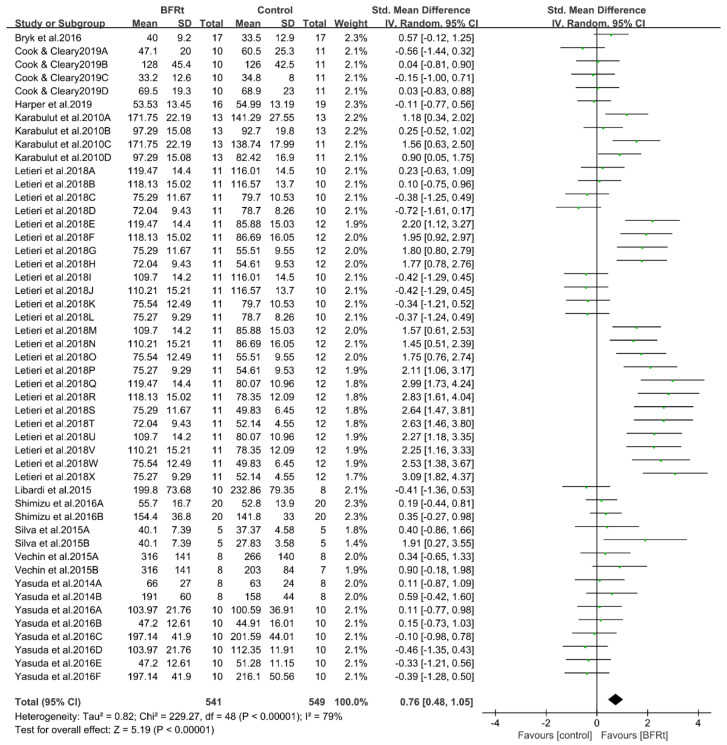
Pooled outcomes of the effect of BFRt intervention on lower-limb muscle strength. Different letters for the same study represent different muscle strength assessment methods [19,22,29,30,31,32,33,34,35,36,37].

Eleven studies compared differences in lower extremity muscle strength between the BFR exercise group and the control group. The combined SMD showed high heterogeneity (I^2^ = 79% and *p* < 0.001). Therefore, the subsequent meta-analysis was conducted using the random effect model. As shown in Figure 4, comprehensive meta-analysis results showed that BFRt could effectively improve lower-limb muscle strength (SMD = 0.76, 95% CI: 0.48 to 1.05, and *p* < 0.001). This finding suggests that post-middle-aged adults who are unable to perform high intensity resistance training may gain muscle strength through BFRt.

We conducted a meta-regression analysis to determine the correlation between intervention variables (i.e., frequency, comparator intervention group, cuff pressure, duration, exercise mode) and muscle strength. One of our key findings was that the changes in muscle strength were significantly correlated to the training frequency (coef. = 0.84, 95% CI = 0.11 to 1.58, and *p* = 0.026) and comparator intervention group (coef. = 0.82, 95% CI = 0.52 to 1.12, and *p* = 0.000). Moreover, the duration (coef. = 0.03, 95% CI = −0.06 to 0.13, and *p* = 0.442), cuff pressure (coef. = −0.0008, 95% CI = −0.006 to 0.007, and *p* = 0.800), and exercise mode (coef. = −0.03, 95% CI = −0.25 to 0.30, and *p* = 0.839) were not significantly correlated with the change in muscle strength (Table 2).

**Figure 5:**
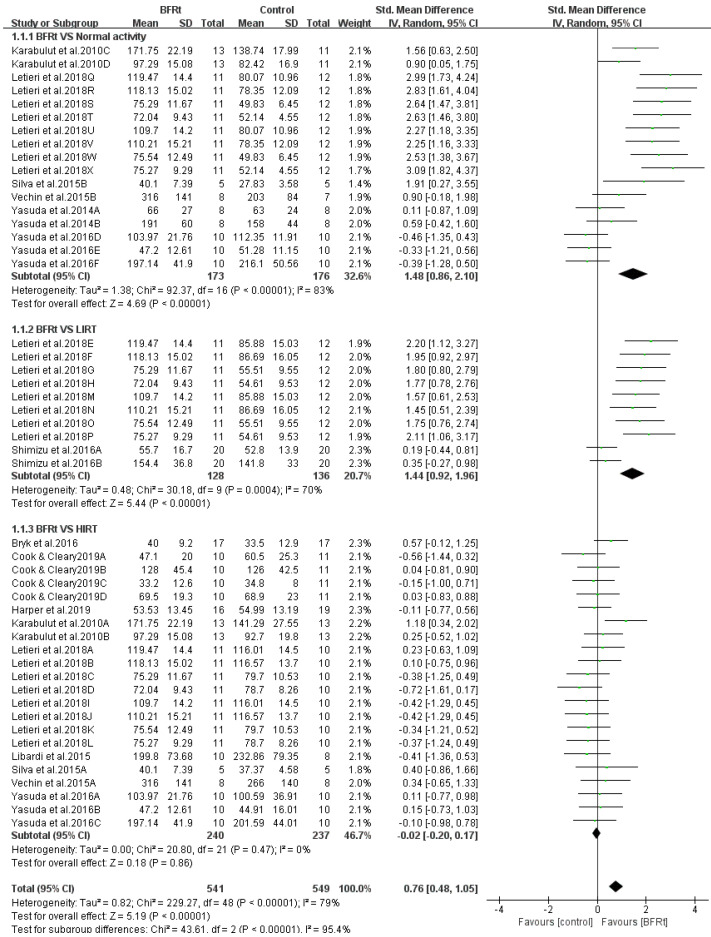
Subgroup analysis of the effect of BFRt intervention on lower-limb muscle strength. Different letters for the same study represent different muscle strength assessment methods [19,22,29,30,31,32,33,34,35,36,37].

Subgroup analysis was performed to identify the beneficial variables of intervention (i.e., frequency and comparator intervention group) on muscle strength gain in post-middle-aged adults. As shown in Figure 5, BFRt resulted in greater muscle strength gain when compared to normal activity (SMD = 1.48, 95% CI = 0.86 to 2.10, *p* < 0.01, and I^2^ = 83%). Greater muscle strength gain was also noticed when compared to LIRT alone (SMD = 1.44, 95% CI = 0.92 to 1.96, *p* < 0.01, and I^2^ = 70%). Interestingly, no statistical difference in muscle strength gain was observed when BFRt was compared to HIRT (SMD = −0.02, 95% CI = −0.20 to 0.17, *p* < 0.01, and I^2^ = 0). In addition, tests for subgroup differences revealed significant differences among three subgroups (*p* < 0.00001) (Figure 5).

**Figure 6:**
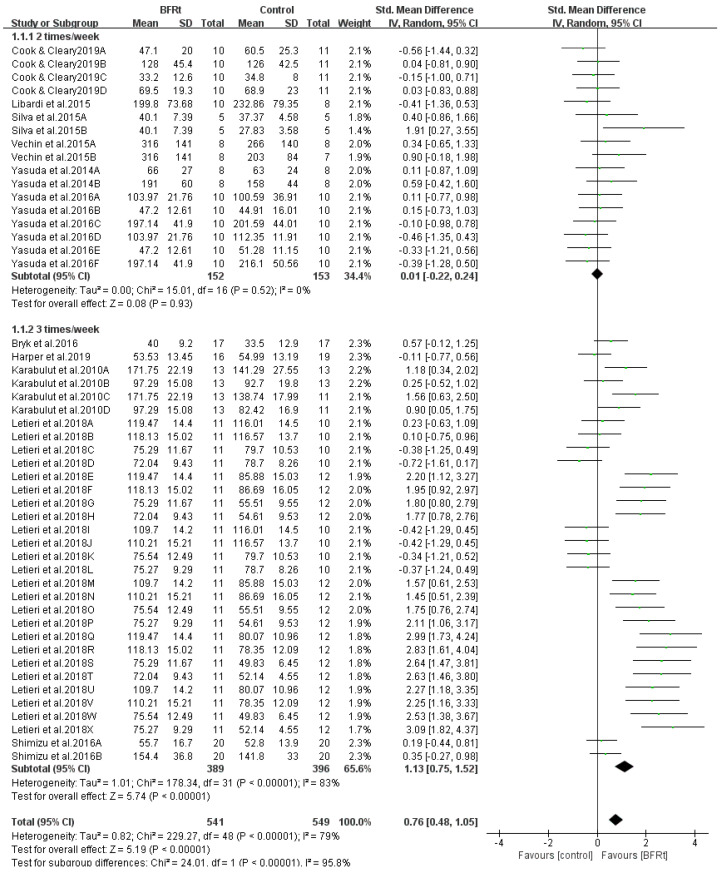
Subgroup analysis of the effect of BFRt training frequency on lower-limb muscle strength. Different letters for the same study represent different muscle strength assessment methods [19,22,29,30,31,32,33,34,35,36,37].

The results of the subgroup analysis for training frequency (three times/wk, two times/wk) are shown in Figure 6. The muscle strength gain was found when the training frequency was three times a week (SMD = 1.13, 95% CI = 0.75 to 1.52, *p* < 0.001, and I^2^ = 83%). In contrast, no muscle strength gain was noticed when training frequency was two times a week (SMD = 0.01, 95% CI = −0.22 to 0.24, *p* = 0.93, and I^2^ = 0). Tests for subgroup differences revealed a significant difference between the groups (*p* < 0.00001), which emphasizes the correlation between exercise frequency and muscle strength gains (Figure 6). In addition, to address the influence of training volume on muscle strength, we performed another subgroup analysis. We found significant strength gain when training frequency was three times per week in volume-equated trials (Supplementary Figure S1).

### 3.5. Effects of BFRt on Lower-Limb Muscle Mass in Post-Middle-Aged Adults

**Figure 7:**
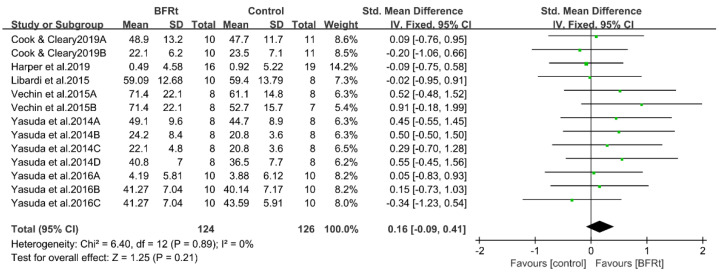
Pooled outcomes of the effects of the BFRt intervention on lower-limb muscle mass. Different letters for the same study represent different muscle mass assessment methods [19,22,32,33,35,36].

Another outcome measure assessed in this study was muscle mass. A total of six studies reported muscle mass data. The data were determined by cross-sectional area (five studies), and muscle thickness/body lean mass (one study) methods. The meta-analysis results showed no statistical difference in lower-limb muscle mass between the BFRt group and the control group (SMD = 0.16, 95%CI = −0.09 to 0.41, *p* = 0.21, and I^2^ = 0) (Figure 7).

## 4. Discussion

The main purpose of this meta-analysis was to compare the effects of BFRt on lower-limb muscle strength and mass in post-middle-aged adults. The included studies reported the effects of BFRt on lower-limb muscle strength or mass in adults and compared these with other intervention training methods without BFR. The comprehensive results showed that BFRt can significantly improve the lower-limb muscle strength of post-middle-aged adults when compared with other interventions; however, there was no statistical difference in muscle mass improvement. The meta-regression analysis showed that the improvement in muscle strength was associated with training frequency and a comparator intervention group. However, there was no significant correlation with training duration, cuff pressure, and exercise mode. Subgroup analysis results further indicated that greater muscle strength gain was achieved with BFRt when compared to LIRT and normal activity, and strength gain comparisons between BFRt and HIRT were not statistically different. In addition, there was a strong correlation between training frequency and muscle strength gain. Our findings suggest that a frequency of three times/wk is better than a frequency of two times/wk for muscle strength gain. These findings suggest that BFR combined with LIRT can improve lower extremity muscle strength in post-middle-aged adults, and frequency is the considerable variable.

The results of our meta-analysis of 11 RCTs showed greater muscle strength gain with BFRt when compared to LIRT or normal activity, but no statistical difference between BFRt and HIRT was seen. Muscle strength gain after BFRt was also reported in recent meta-analyses [25,26], but these studies did not address the influence of training frequency. A research study reported that both BFRt and HIRT for 12 weeks can improve strength gain in older adults, and the benefits of BFRt and HIRT were not statistically different [38]. The reasons for that BFRt achieves similar muscle benefits to HIRT may be due to the nerve fatigue and muscle tissue hypoxia caused by vascular occlusion. The increased accumulation of metabolites in BFRt can cause neuromuscular fatigue more rapidly through metabolic stimulation of the afferent nerves in groups III and IV [39] or in the suppression of the transbridge circulation [40], which promotes muscle growth. As BFRt leads to limited oxygen supply to the muscle, which may lead to the prerecruitment of anaerobic fast muscle fibers in order to maintain muscle strength [41], BFRt is thought to stimulate a larger number of muscle fibers. These results support the notion that BFRt produces the same benefit level as HIRT in post-middle-aged adults.

To the best of our knowledge, this is the first systematic review and meta-analysis to address the effect of BFRt on lower-extremity muscle strength in post-middle-aged adults and to explore the importance of training frequency. We found that the training frequency significantly correlated with improved muscle strength gain. During resistance training, frequency significantly affects the muscle strength, with a higher training frequency producing more strength and muscle mass [42,43,44]. Studies have shown that exercise-induced increases in muscle protein synthesis last approximately 24–48 h [45,46]. In addition, a higher training frequency can evenly distribute training volume throughout the week, reducing fatigue [47]. Performing more sets per session while using a lower training frequency may reduce the time spent in a positive net protein balance due to the fact that the large number of sets performed within a given session may exceed the ‘anabolic limit’, which, thus, result in wasted sets [48]. Increasing the number of muscle group movements in a single session does not necessarily provide greater muscle strength gains, as there may be a threshold for each training session [49]. For this reason, increasing the number of sets performed in a given training session may simply prolong fatigue without providing a greater increase in muscle gains [48]. For people without training experience, high frequency means more opportunities to contact and practice. The proficiency of the movements will increase accordingly, and the gains will be relatively significant. The study of Fujita et al. [50] showed that after 6 days (with 12 repetitions) of LIRT, quadriceps CSA and volume increased by 3.5 and 3.0%, respectively. The muscle mass and strength changes were similar to several weeks of high-intensity resistance training. Interestingly, the blood levels of creatine kinase, myoglobin, and interleukin-6 remained unchanged throughout the training process; therefore, no apparent muscle damage related to sports training was found. Abe et al. [51] also demonstrated that skeletal muscle hypertrophy and strength increase occurred after a high frequency (2 weeks, twice a day at 6 days per week) of BFRt. Our findings on volume-equated studies showed strength gain only with a training frequency of three times a week, and not with a frequency of two times a week. We considered these findings to be explained by muscle adaptation caused by the high frequency of BFRt; however, the participants were all healthy young people, and the mechanism of the above research should be verified in a post-middle-aged adult population.

Previous studies have suggested that the underlying mechanisms of similar muscle adaptation induced by BFR when compared to HIRT may be due to hormonal responses, intracellular signaling pathways, satellite cell activity, and fiber-type recruitment patterns [52,53,54,55]. However, it is important to note that recent studies have shown BFRt-induced muscle hypertrophy was not associated with hormonal response in young adults [56]. After the training of skeletal muscle, the increase in surrounding metabolites causes cell swelling, which changes the pressure gradient inside and outside the cell membrane and is conducive to the blood flow to the muscle cell again (i.e., blood reperfusion). This is such that the corresponding osmotic pressure sensor on the cell membrane starts the signal response, activates the protein synthesis pathway, and finally contributes to muscle strength growth [57,58]. The recruitment of fast-twitch muscle fibers by metabolic stress appears to be an important factor in the gain of muscle strength. The fast-twitch muscle fibers consume less oxygen compared to slow-twitch muscle fibers, produce greater muscle strength, and are typically recruited during high-intensity exercise. However, the metabolites produced by BFR and the hypoxic environment allow fast-twitch muscle fibers to be recruited and promote muscle strength gain [59,60].

Limitations: Our meta-analysis included research papers from high-quality sources, but there are still some limitations that should be mentioned: (1) The number of studies on muscle mass were few; thus, we could not conduct regression analysis and subgroup analysis. (2) The age of the participants in the trial ranged from 50 to 90 years. Although the age range appears to be wide, this may not influence the final outcome of our study. (3) This study collected data from trials published in English, which may limit the comprehensiveness of our findings to a certain extent. (4) Lastly, we studied the effects of BFRt intervention on muscle adaptation in post-middle-aged adults without considering the influence of region, gender, and/or body mass index on the training effect. Further analyses in the future are necessary to overcome these limitations.

## 5. Conclusions

Our results revealed that BFRt increases lower-limb muscle strength in post-middle-aged adults. We further report that BFRt can achieve similar muscle strength gains when compared to HIRT without BFR. Therefore, BFRt is an effective intervention among adults to improve their muscle strength. Our results further show that there is a correlation between muscle strength gain and training frequency. When the training frequency was three times a week, muscle strength was effectively improved among adults. Muscle strength gain is essential among post-middle-aged adults to maintain their daily activities as they get older. Therefore, training frequency should be considered when designing BFRt interventions for post-middle-aged adults.

## Figures and Tables

**Table 1:** Characteristics of the included studies.

Study	Country	Age	Gender, M/F	Groups/Sessions	Exercise Mode	Duration (wk)	Frequency (t/wk)	Sets × Repetitions	Cuff Pressure (mm Hg)	Cuff Width (cm)	Muscle Strength/Mass
Bryk et al., 2016 [30]	Brazil	30% 1RM-BFR, 62.3 ± 7; 70% 1RM, 60.4 ± 6.7	34/0	30% 1RM-BFR, N = 17; 70% 1RM, N = 17	quadriceps exercise	6	3	30% 1RM-BFR 3 × 10; 70% 1RM 3 × 10	200	NR	Quadriceps strength
Cook & Cleary 2019 [32]	USA	67–90	9/12	30% 1RM-BFR, N = 10; 70% 1RM, N = 11	Knee flexion, leg press, knee extension	12	2	30% 1RM-BFR 3 × 10; 70% 1RM 3 × 10	184 ± 25	6	Knee extension (10RM, MVC), Knee flexion (10RM, MVC); Quadriceps CSA, Hamstrings CSA
Harper et al., 2019 [33]	USA	≥60	NR	20% 1RM-BFR, N = 16; 60% 1RM, N = 19	leg press, leg extension, calf flexion, leg curl,	12	3	NR	0.5 (SBP) + 2 (thighcircumference) + 5	NR	knee extensor Strength torque; Lower body lean mass
Karabulut et al., 2010 [34]	USA	56.8 ± 0.6	37/0	20% 1RM-BFR, N = 13; 80% 1RM, N = 13; normal activity, N = 11	leg press, leg extension	6	3	20% 1RM-BFR 30,15,15; 80% 1RM 3 × 8;	205.4 ± 4.3	NR	Leg press strength, Leg extension strength
Libardi et al., 2015 [19]	Brazil	64.7 ± 4.1	NR	20–30% 1RM-BFR, N = 10; 70–80% 1RM, N = 8	leg press	12	2	20–30% 1RM-BFR 30,15,15,15; 70–80% 1RM 4 × 10	67 ± 8.0	17.5	Leg press 1RM; Quadriceps CSA
Shimizu et al., 2016 [37]	Japan	71 ± 4	33/7	20% 1RM-BFR, N = 20; 20% 1RM, N = 20	leg extension, leg press	4	3	20% 1RM-BFR 3 × 20; 20% 1RM 3 × 20	100% femoral SBP	10	Leg extension 1RM, Leg press 1RM,
Silva et al., 2015 [31]	Brazil	62.2 ± 4.53	0/15	30% 1RM- BFR, N = 5; 80% 1RM, N = 5; normal activity, N = 5	knee extension (right, leg)	12	2	30% 1RM-BFR 4 × (7.0 ± 3.38); 80% 1RM 4 × 8.0 ± 2.0	104.20 ± 7.88	18	Leg extension-R 1RM
Vechin et al., 2015 [22]	Brazil	64.04 ± 3.81	14/9	20–30% 1RM-BFR, N = 8; 70–80% 1RM, N = 8; normal activity, N = 7	leg press	12	2	20–30% 1RM-BFR 30,15,15,15; 70–80% 1RM, 4 × 10	71 ± 9	18	Leg press 1RM; Quadriceps CSA
Yasuda et al., 2016 [35]	Japan	61–86	0/30	35–45% 1RM-BFR, N = 10; 70–90% 1RM, N = 10; normal activity, N = 10	bilateral squat, knee extension	12	2	35–45% 1RM-BFR 30,15,15,15; 70–90% 1RM 13, 13 (at 1st–12th training session) or 12 (at 13th–24th training session	161 ± 12	5	Knee extension, (1RM, MVC), Leg press 1RM; Quadriceps CSA, Muscle thickness of mid-thigh
Yasuda et al., 2014 [36]	Japan	61–78	5/11	20–30% 1RM-BFR, N = 8; normal activity, N = 8	NR	12	2	NR	120–270	NR	Knee extension 1RM, Leg press 1RM; Quadriceps CSA, Adductors CSA, Hamstrings CSA, Gluteus maximus CSA
Letieri et al., 2018 [29]	Brazil	68.8 ± 5.09	0/56	20–30% 1RM- BFR- high occlusion pressure, N = 11; 20–30% 1RM-BFR-low occlusion pressure, N = 11; 70–80% 1RM, N = 10; 20–30% 1RM, N = 12; normal activity, N = 12	Squat, Leg Press, Knee Extension, Leg Curl	16	3	20–30% 1RM- BFR-high occlusion pressure 3–4 × 15; 20–30% 1RM- BFR- low occlusion pressure 3–4 × 15; 70–80% 1RM 3–4 × 6–8; 20–30% 1RM 3–4 × 15	20–30% 1RM-BFR-H 185.75 ± 5.45 20–30% 1RM-BFR-L 105.45 ± 6.5	NR	Peak Torque Extension (right, left), Peak Right Flexion (right, left)

Note: BFR: blood flow restriction; RM: repetitions maximum; MVC: the largest voluntary contraction; CSA: the cross-sectional area of muscle; NR: unreported; SBP: systolic blood pressure; M/F: male/female; t/wk: times/week.

**Table 2:** Meta-regression analysis of the trial interventions.

Experimental Intervention	Coefficient	Standard Error	T-Value	*p*-Value	[95% Conf. Interval]	
Comparator intervention group	0.820633	0.1485629	5.52	0.000	0.5198832	1.121383
Exercise mode	0.0279026	0.136192	0.20	0.839	−0.2478037	0.3036089
Cuff pressure	0.0008191	0.00321	0.26	0.800	−0.0056792	0.0073174
Duration (wk)	0.0348827	0.0448527	0.78	0.442	−0.0559168	0.1256822
Frequency (t/wk)	0.8428523	0.3637526	2.32	0.026	0.1064737	1.579231

## Data Availability

Not applicable.

## Supplementary Materials

The following supporting information can be downloaded at: https://www.mdpi.com/article/10.3390/ijerph192315691/s1, Figure S1: Forest plot for volume equated studies in different frequencies.
