# Supplementary material for: Effects of Blood Flow Restriction Combined with Low-Intensity Resistance Training on Lower-Limb Muscle Strength and Mass in Post-Middle-Aged Adults: A Systematic Review and Meta-Analysis
Source: Int J Environ Res Public Health. 2022 Nov 25;19(23):15691. doi: 10.3390/ijerph192315691 (PMC9735845; doi:10.3390/ijerph192315691)
Supplement: Supplementary file 1 [file ijerph-19-15691-s001.zip › ijerph-1971195-supplementary.pdf]

Supplementary Materials

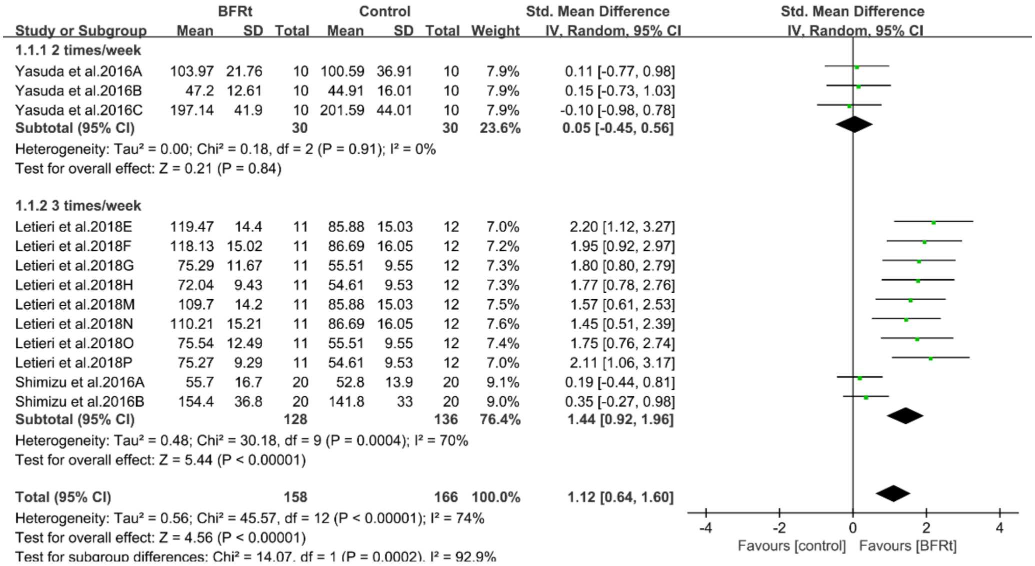

Figure S1. Forest plot for volume equated studies in different frequencies [29,35,37].
